# The effects of botulinum toxin type A on the trigeminal TRPV1 containing neurons innervating the dura mater of rat

**DOI:** 10.1186/1129-2377-14-S1-P82

**Published:** 2013-02-21

**Authors:** T Shimizu, M Shibata, H Toriumi, T Iwashita, M Funakubo, H Sato, T Kuroi, T Ebine, K Koizumi, N Suzuki

**Affiliations:** 1 Keio University, School of Medicine, Japan; 2 Keio University, School of Medicine, Japan

## Background

Botulinum toxin type A (BTX-A) has been used for prophylactic treatment in chronic migraine. However, the precise mechanism of its action is obscure. We previously reported that the injection of BTX-A into the facial ophthalmic nerve region reduced the number of the TRPV1-immunoreactve (IR) neurons in the trigeminal ganglion (TG). The dura mater, known as an important site of headache generation, is densely innervated by trigeminal nociceptors. We have recently demonstrated the existence of TRPV1-IR nerve fibers in the dura mater that originate in TG. In this study, we explored the effect of BTX-A on the number of the TRPV1-IR TG neurons innervating the dura mater.

## Methods

Six Sprague-Dawley rats were used. The retrograde tracer, true blue (TB), was applied to the dura mater. Seven days after the tracer application, 0.5 ng/kg BTX-A was injected into the left side of the face in three animals, and three control animals were injected with saline at the same location. After 7 days, TGs were dissected out and immunostained with an anti-TRPV1 antibody. For analysis, we calculated the ratio of the TRPV1-IR cells in TB accumulated neurons. Results In the control animals, the proportion of TRPV1-IR-containing neurons that were also TB-positive was 27 % (n = 372 neurons from 3 animals). In the BTX-A treated animals, tracer accumulation and TRPV1-IR were also observed. However, the number of TRPV1-IR neurons retrogradely labeled with true blue was reduced. The proportion of TRPV1-IR cells in the BTX-A treated animals was 11 % (n = 504 neurons from 3 animals), and this was significantly decreased compared to the control group (Student's t-test, p < 0.0001).

## Conclusion

Our results indicated the possibility that BTX-A may reduce the expression of the TRPV1 receptor in neurons of the TG innervating the dura mater, which may account for its alleviating action against headache disorders.

